# Supplementary material for: Comprehensive promotion of iPSC-CM maturation by integrating metabolic medium with nanopatterning and electrostimulation
Source: Nat Commun. 2025 Mar 21;16:2785. doi: 10.1038/s41467-025-58044-6 (PMC11928738; doi:10.1038/s41467-025-58044-6)
Supplement: Supplementary file 1 — Supplementary Information [file 41467_2025_58044_MOESM1_ESM.pdf]

Supplementary information

Comprehensive promotion of iPSC-CM maturation by integrating metabolic medium with nanopatterning and electrostimulation

Wener Li[1], Xiaojing Luo[1], Anna Strano[1], Shakthi Arun[1], Oliver Gamm[1], Mareike S. Poetsch[1], Marcel Hasse[1], Robert-Patrick Steiner[1], Konstanze Fischer[1], Jessie Pöche[1], Ying Ulbricht[1], Mathias Lesche[2], Giulia Trimaglio[3,4], Ali El-Armouche[1], Andreas Dahl[2], Peter Mirtschink[3], Kaomei Guan*[1], Mario Schubert*[1]

[1] Institute of Pharmacology and Toxicology, Technische Universität Dresden, 01307 Dresden, Germany, kaomei.guan@tu-dresden.de and mario.schubert1@tu-dresden.de

[2] DRESDEN-concept Genome Center, Center for Molecular and Cellular Bioengineering, Technische Universität Dresden, 01307 Dresden, Germany

[3] Institute of Clinical Chemistry and Laboratory Medicine, Department of Clinical Pathobiochemistry, University Hospital Dresden, 01307 Dresden, Germany

[4] National Center for Tumor Diseases, Partner Site Dresden, 01307 Dresden, and German Cancer Research Center, 69120 Heidelberg, Germany

This file includes:

Supplementary figures

Supplementary tables

Supplementary Figure 1

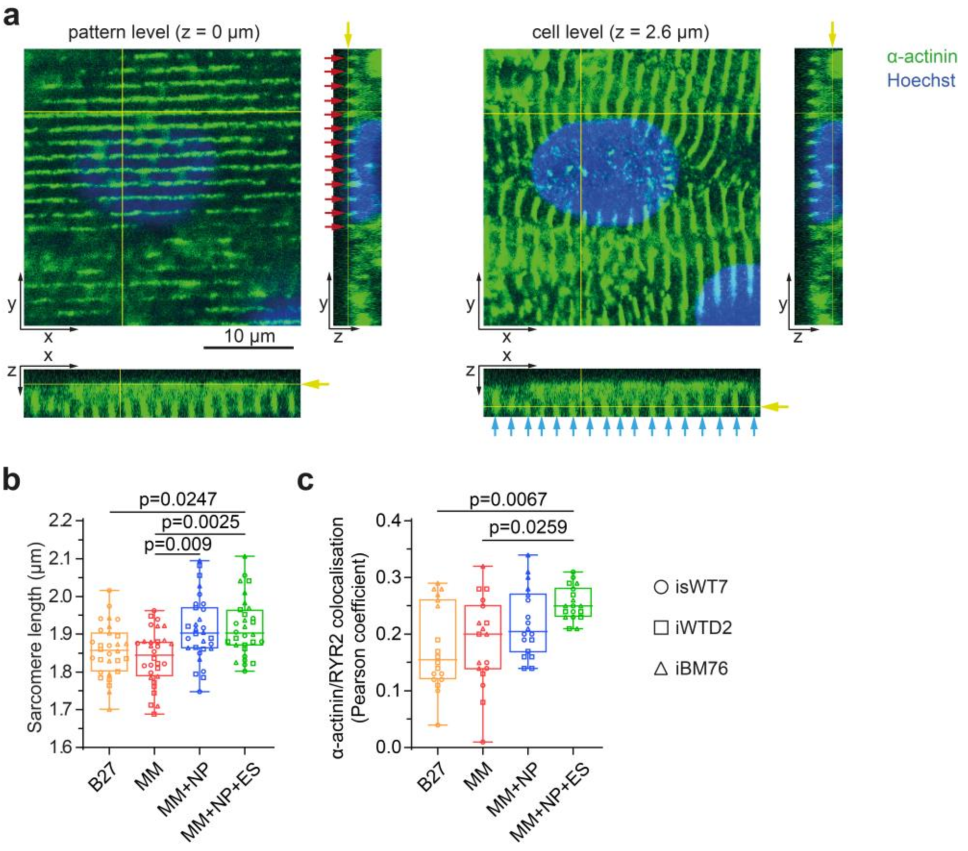

Supplementary Fig. 1: Analysis NP orientation, sarcomere length and α-actinin-RYR2 colocalisation. a Detection of iPSC-CMs stained for α-actinin and nuclei and the NP on the coverslip at different z levels using confocal microscopy. Orthogonal projections of two images illustrating the z level of the NP surface (left) and the cell (right). Yellow lines and arrows indicate the position of the projection. Red arrows mark the grooves of the NP surface (left, y-z projection), and blue arrows mark the z-disks (right, x-z projection). To analyse sarcomere alignment, the NP direction was defined as 0° in the MM+NP and MM+NP+ES groups, whereas 0° was randomly defined in the B27 and MM groups. The z-disk orientation (stained with α-actinin) and the direction of the elongated nuclei (stained with Hoechst 33342) were calculated with respect to 0° using Cell Profiler software. **b** Sarcomere length of iPSC-CMs. n=30 cells/group from 3 iPSC lines (10 cells/line). **c** Quantification of α-actinin/RYR2 colocalisation. n = 18 images/group from 3 iPSC lines (6 images/line). Source data are provided as a Source Data file. Statistical analysis was performed using Kruskal-Wallis test with Dunn's multiple comparison test. Data are presented as box plots indicating median (middle line), 25th, 75th percentile (box) and min and max data points (whiskers) in **b** and **c**.

Supplementary Figure 2

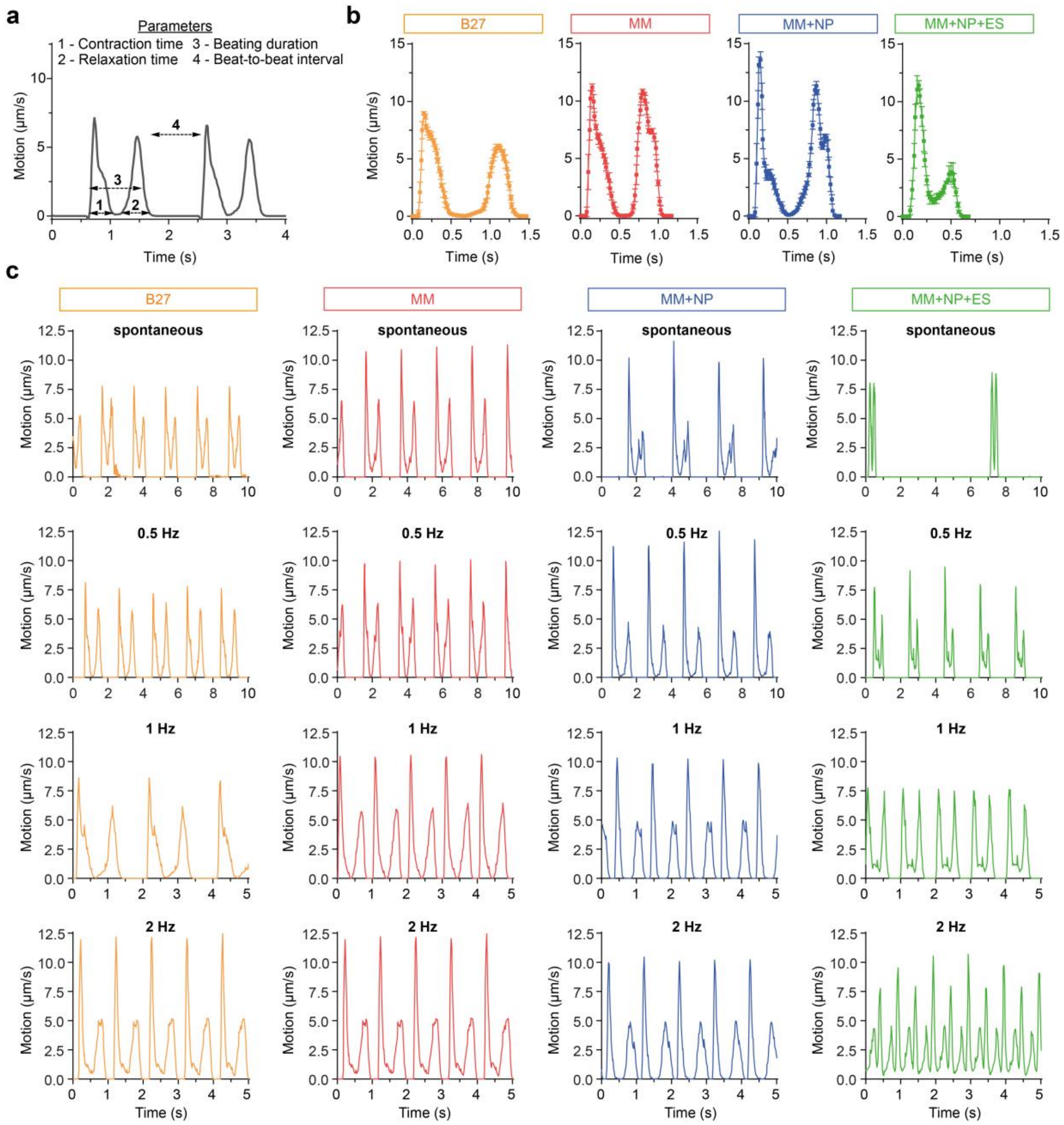

Supplementary Fig. 2: Movie-based motion analysis of iPSC-CMs on day 42. a Schematic diagram of two beat traces showing how the indicated parameters were analysed. **b** Averaged beating traces from iPSC-CMs at 0.5 Hz stimulation for the four groups. **c** Beating traces from the spontaneous beating and in the presence of increased pacing frequencies.

Supplementary Figure 3

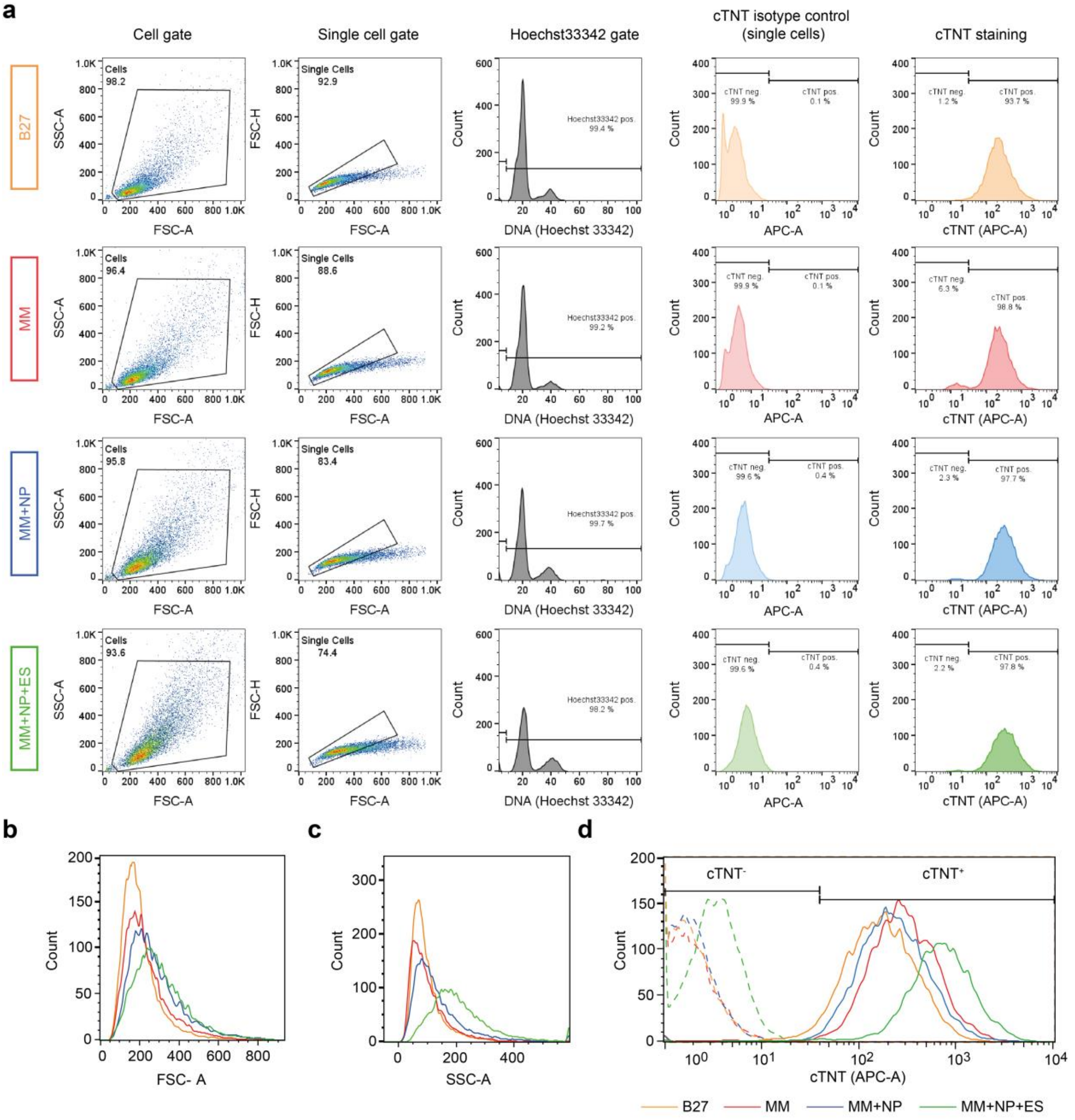

Supplementary Fig. 3: Gating strategy used for analysis of flow cytometry data. a Representative data of iPSC-CMs derived from the isWT7.22 line at day 42. **b**-**c** Histogram of FSC-A (**b**) and SSC-A (**c**) in the cTNT-positive CM populations. **d** Proportion of cTNT-positive cells.

Supplementary Figure 4

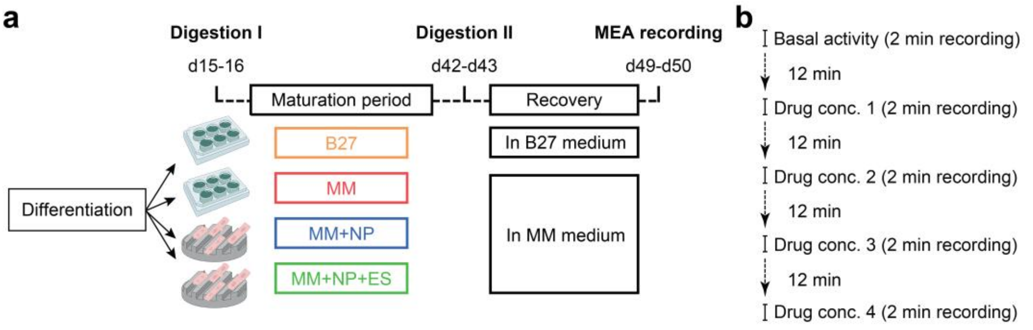

c Number of quiescent cultures

	Verapamil				E-4031				Isoprenaline			
	B27	MM	MM+NP	MM+NP +ES	B27	MM	MM+NP	MM+NP +ES	B27	MM	MM+NP	MM+NP +ES
Cultures analysed (n wells/N batches)	17/3	17/3	18/3	18/3	10/2	12/2	11/2	12/2	18/3	23/4	23/4	24/4
Baseline	0	0	0	0	0	0	0	0	0	0	0	0
1 nM	0	0	0	0	0	0	0	0	0	0	0	0
10 nM	0	0	0	0	0	0	0	0	0	0	0	0
100 nM	5	0	0	0	n.d.	n.d.	n.d.	n.d.	0	0	0	0
1 μM	17	6	6	0	n.d.	n.d.	n.d.	n.d.	0	0	0	0

d Number of arrhythmic cultures

	Verapamil				E-4031				Isoprenaline			
	B27	MM	MM+NP	MM+NP +ES	B27	MM	MM+NP	MM+NP +ES	B27	MM	MM+NP	MM+NP +ES
Baseline	0	0	0	0	0	0	0	0	0	0	0	0
1 nM	2	1	0	1	0	0	2	1	0	0	1	2
10 nM	1	0	2	0	0	2	2	1	0	0	0	0
100 nM	0	0	0	1	n.d.	n.d.	n.d.	n.d.	0	0	0	1
1 μM	--	3	0	0	n.d.	n.d.	n.d.	n.d.	0	0	0	0

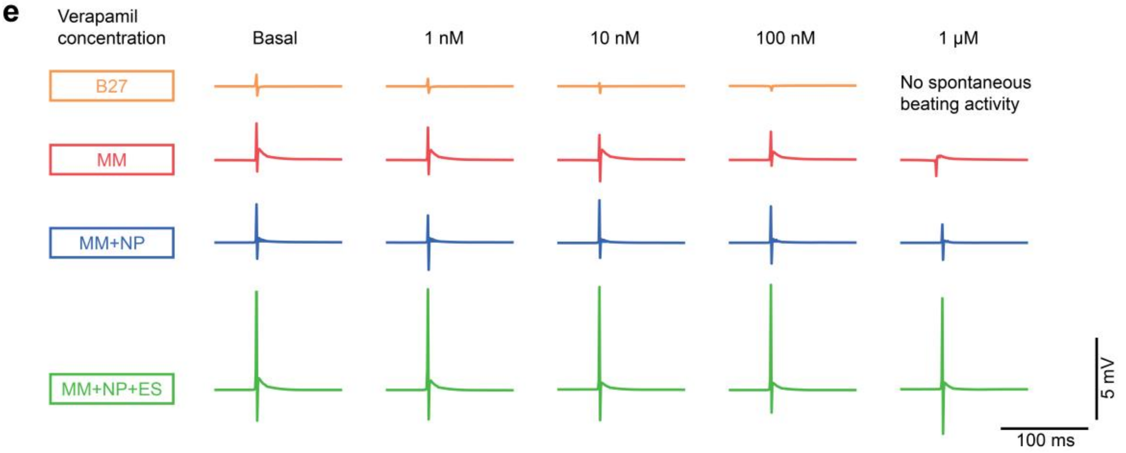

Supplementary Fig. 4: Drug response of iPSC-CMs using MEA. a Experimental scheme. Created in BioRender. Li, W. (2025) https://BioRender.com/m69m593. **b** Sequential drug adding protocol. **c**,**d** Number of quiescence (**c**) and arrhythmic (**d**) cultures from n=17 (B27, MM) and 18 (MM+NP, MM+NP+ES) from 3 independent experiments of 2 iPSC lines for verapamil; n=10 (B27), 12 (MM), and 11 (MM+NP, MM+NP+ES) from 2 independent experiments for E-4031; and n=18 (B27), 23 (MM, MM+NP), and 24 (MM+NP+ES) cultures from 3 (B27) or 4 (MM, MM+NP, MM+NP+ES) independent experiments of 2 iPSC lines for isoprenaline. n.d., not done. **e** Representative field potential traces illustrating the change in spike amplitude induced by verapamil.

Supplementary Figure 5

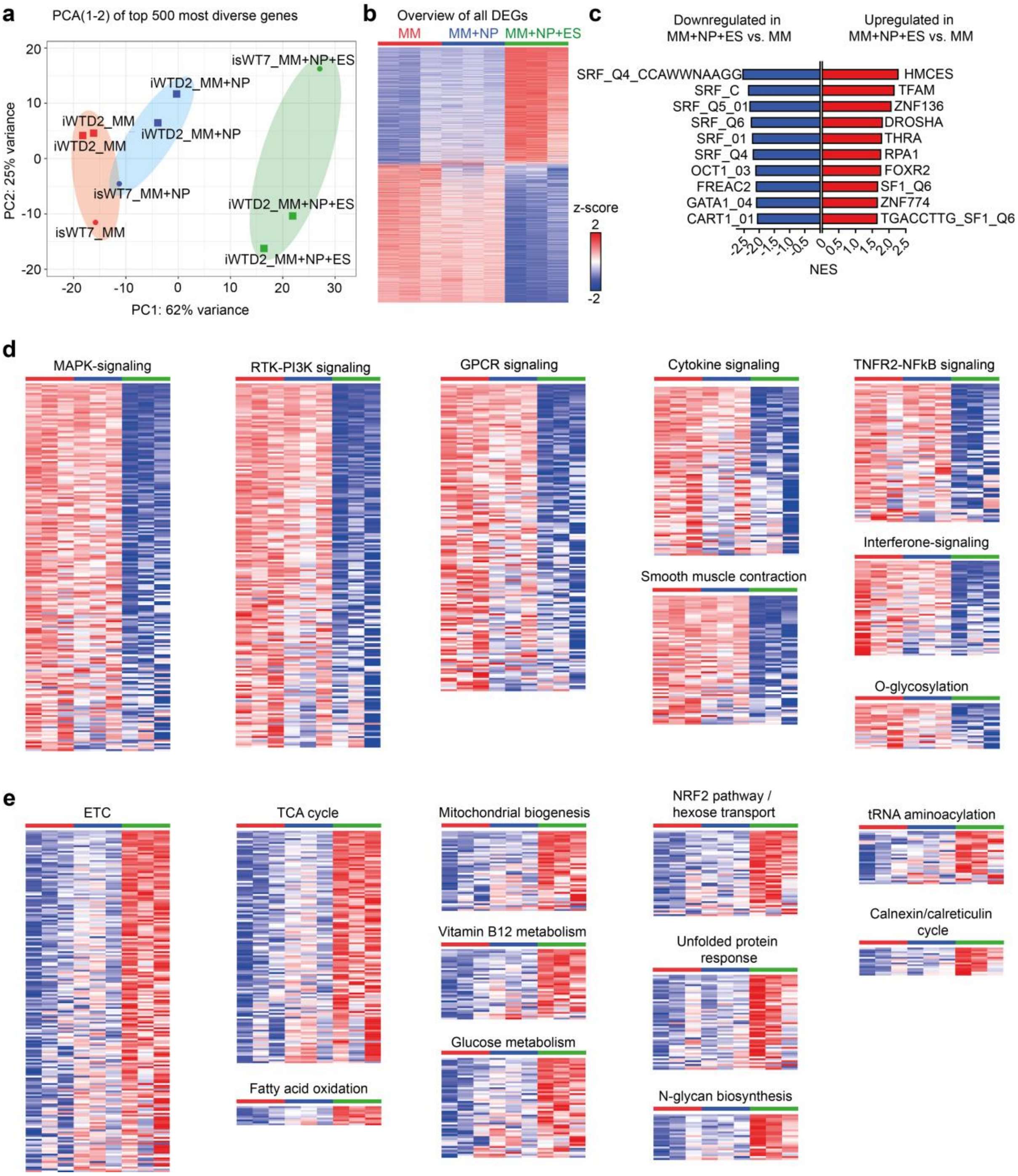

Supplementary Fig. 5: RNA sequencing analysis. a Principal component analysis (PCA) based on the top 500 most diverse genes. **b** Heatmap of all differentially expressed genes (DEGs) between MM+NP+ES vs. MM, MM+NP vs. MM and MM+NP+ES vs. MM+NP. **c** Top 10 up- and downregulated transcription factor targets in iPSC-CMs from MM+NP+ES vs. MM conditions (NES, normalised enrichment score). Source data are provided as a Source Data file. **d**,**e** Heatmaps of core enrichment genes in downregulated (**d**) and upregulated (**e**) pathway clusters in iPSC-CMs from MM+NP+ES vs. MM groups. The colour scale represents z-score.

Supplementary Figure 6

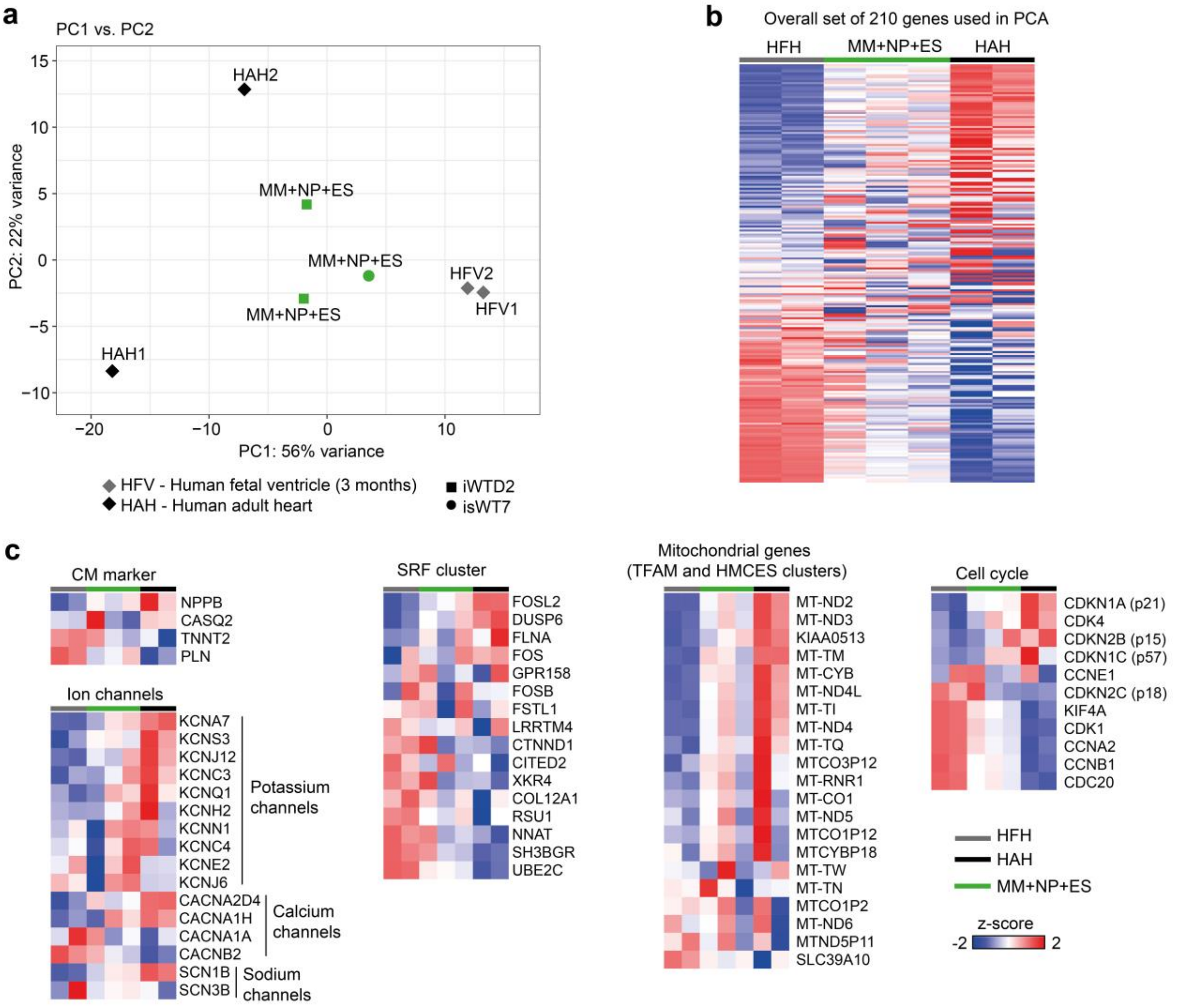

Supplementary Fig. 6: RNA sequencing analysis. a Principal component analysis of MM+NP+ES group in comparison to published RNA-seq datasets from human fetal ventricle (HFV) and human adult heart (HAH) samples, based on 210 manually selected genes including CM markers, ion channels as well as SRF target genes, mitochondrial genes and cell cycle genes that were regulated in MM+NP+ES group in comparison to MM (gene list and dataset see Supplementary Data 3). **b** Heatmap of all 210 genes. **c** Heatmaps of DEGs ($p<0.05$) between MM+NP+ES and HFV, grouped into categories related to main manuscript. Source data are provided as a Source Data file.

Supplementary Figure 7

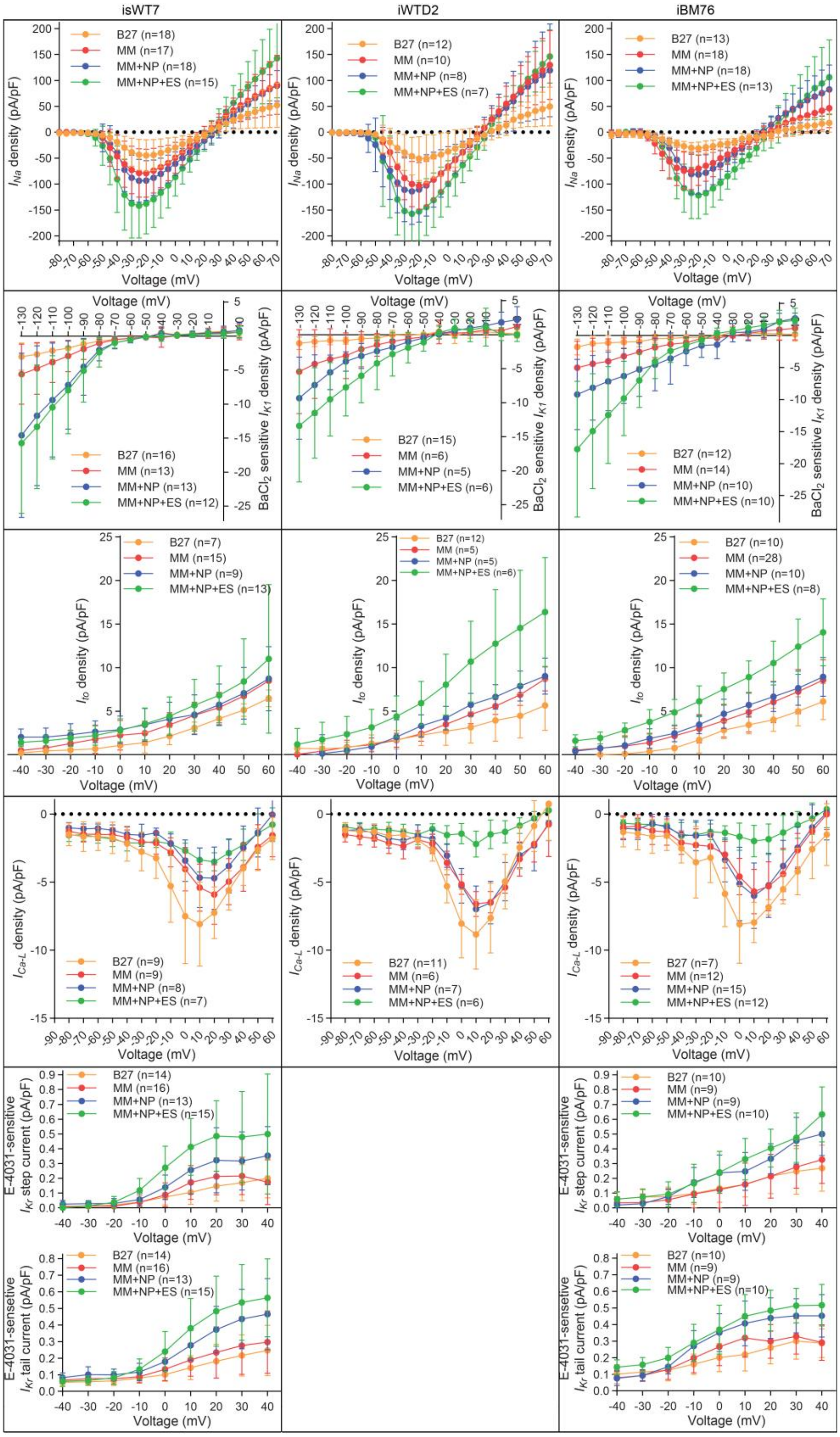

Supplementary Fig. 7: iPSC line-based plotting of patch clamp measurements of I_{Na}, $I_{Ca\text{-}L}$, I_{to}, I_{K1}, and I_{Kr}. Data from 3 iPSC lines (isWT7, iWTD2 and iBM76) for I_{Na}, $I_{Ca\text{-}L}$, I_{to}, and I_{K1}, and 2 iPSC lines (isWT7 and iBM76) for I_{Kr}. Two differentiation experiments of each cell line were performed. n≥3 cells per differentiation and group were included. No significant differences among three iPSC lines were observed. Data are presented as mean ± SD. Source data are provided as a Source Data file, as for Figures 2, 3 and 4.

Supplementary Table 1: Formulation of media used in this study compared to the published medium from Feyen et al. 2020

Component	B27 medium	Maturation medium	Feyen et al. 2020	DMEM+B27
Base medium	RPMI 1640 (Gibco, 72400021)	DMEM without glucose (Thermo Fisher Scientific 11966025)	DMEM without glucose (Thermo Fisher Scientific 11966025)	DMEM without glucose (Thermo Fisher Scientific 11966025)
Supplement	1x B27 supplement (Gibco, 17504044)	1x B27 supplement minus insulin (Gibco A1895601)	1x B27 supplement (Gibco, 17504044)	1x B27 supplement (Gibco, 17504044)
Human insulin (Sigma I9278)	700 nM in B27	50 nM	700 nM in B27	700 nM in B27
D-glucose	11.1 mM in RPMI 1640	7 mM	3 mM	11.1 mM
L-lactate (Sigma Aldrich, 71718)	-	0.8 mM	10 mM	-
L-ascorbic acid-2-phosphate (Sigma Aldrich, A8960)	-	0.5 mM	0.5 mM	-
Taurin (Sigma Aldrich, T0625)	-	2 mM	2 mM	-
Creatine-monohydrate (Sigma Aldrich, C3630)	-	5 mM	5 mM	-
L-Carnitine - hydrochloride (Sigma Aldrich, C0283)	-	1.6 mM	2 mM	-
Calcium	0.4 mM in RPMI 1640	1.8 mM in DMEM	1.8 mM in DMEM	1.8 mM in DMEM
AlbuMax (Thermo Fisher Scientific, 11020021)	-	0.5%	0.5%	-
NEAA (Gibco, 12084947)	-	1x	1x	-
Knockout Serum Replacement™ (KOSR, Gibco, 10828028)	-	1%	1%	-
Vitamin B12 (Sigma Aldrich, V6629)	3.7 nM in RPMI 1640	0.37 nM	3.7 µM (5 µg/ml)	3.7 nM

Supplementary Table 2: Action potential parameters in the four groups. Data are presented as mean ± SD

	RMP (mV)	Vmax (V/s)	APA (mV)	APD_{90} (ms)
B27	-44.1 ± 9.8 (n=21)	4.2 ± 1.4 (n=21)	70.6 ± 16.7 (n=21)	980.5 ± 393.1 (n=13)
MM	-49.7 ± 8.5 (n=25)	5.0 ± 1.1 (n=25)	78.7 ± 13.9 (n=25)	732.3 ± 256.5 (n=24)
MM+NP	-58.2 ± 7.4 (n=21)	6.6 ± 2.5 (n=21)	88.4 ± 10.8 (n=21)	752.5 ± 375.7 (n=22)
MM+NP+ES	-65.6 ± 8.5 (n=17)	11.0 ± 7.4 (n=14)	97.8 ± 12.7 (n=17)	539.3 ± 186.2 (n=20)

Source data are provided as a Source Data file, as for Figure 2b and 2c.

Supplementary Table 3: Field potential parameters in the four groups. Data are presented as mean ± SD

	CV (cm/s)	Spike amplitude (mV)	Spike slope (V/s)
B27	12.5 ± 5.8 (n=24)	3.2 ± 1.2 (n=24)	-3.6 ± 1.8 (n=24)
MM	22.3 ± 3.7 (n=24)	10.4 ± 4.9 (n=24)	-21.2 ± 11.3 (n=24)
MM+NP	25.6 ± 4.3 (n=24)	10.7 ± 4.8 (n=24)	-23.6 ± 11.1 (n=24)
MM+NP+ES	27.8 ± 7.3 (n=24)	13.6 ± 5.3 (n=24)	-29.1 ± 12.6 (n=24)

Source data are provided as a Source Data file, as for Figure 2g.

Supplementary Table 4: Extracellular and intracellular solutions for automated and manual patch clamp

	Automated patch clamp										Manual patch clamp		
	External solution (For cell catching)	Seal enhancer	***I_{to}***		***I_{Na}***		***I_{Ca-L}***		***I_{K1}***		**AP**	***I_{Kr}***	
in mM			Bath	Pipette	Bath under 25 mM $[Na^+]_o$	Pipette	Bath	Pipette	Bath	Pipette	Bath	Pipette for AP and I_{Kr}	Bath
NaCl	140	130	140	10	25	10		10		10	140		14
Choline chloride													126
TEA-Cl					115		150						
NMDG									140				
KCl	4	4	4	30					5.4	30	5.4	120	5.4
$CaCl_2$	2	10	2		2		2		2		1.8		0.4
$MgCl_2$	1	1	1		1		1		1		1	1	1
CsCl					4	30		30					
KF				90						90			
CsF						90		90					
Glucose	5	5	5		5		5		10		10		10
HEPES	10	10	10	10	10	10	10	10	10	10	10	10	10
EGTA				10		10		10		10		10	
Nifedipine					0.01				0.01				0.01
4-AP													2.5
$CdCl_2$			0.5										0.5
Mg-ATP (freshly)				2		1		1		2		3	
pH adjustment	7.4 with NaOH	7.4 with NaOH	7.4 with NaOH	7.2 with KOH	7.4 with CsOH	7.2 with CsOH	7.3 with CsOH	7.2 with CsOH	7.4 with HCl	7.2 with KOH	7.4 with NaOH	7.2 with KOH	7.4 with NaOH

Supplementary Table 5: Antibodies used for flow cytometry (FC), immunofluorescence staining (IF) and western blot (WB)

Target	Antibody	Specification
**Primary antibodies**		
α-actinin	mouse IgG1, Sigma, A-7811	IF – 1:500
Phalloidin	Alexa Fluor 633, Thermo Fisher Scientific, A-22284	IF – 0.33 µM
cTNT-APC	human IgG1, REAfinity™, Miltenyi Biotec, 130-120-543	FC – 1:50
cTNT	mouse IgG1, Thermo Fisher Scientific, MS-295-P1	FC – 1:500
Cx43	mouse IgG1, BD Bioscience, 610061	IF – 1:1000
GAPDH	mouse IgG1, Santa-Cruz, sc-365062	WB – 1:1000
Isotype control-FITC	human IgG1, REAfinity™, Miltenyi Biotec, 130-113-449	FC – 1:50
Isotype control-APC	human IgG1, REAfinity™, Miltenyi Biotec, 130-113-446	FC – 1:50
Ki67-FITC	human IgG1, REAfinity™, Miltenyi Biotec, 130-117-691	FC – 1:50
RYR2	rabbit IgG, Sigma Aldrich, HAP020028	IF – 1:500
SRF	rabbit IgG, Cell Signaling Technology, #5147	WB – 1:1000
Tom20	mouse IgG2a, Santa Cruz, sc-17764	FC – 1:100

**Secondary antibodies**	
Goat anti-mouse IgG, Alexa Fluor 488 conjugate (Thermo Fisher Scientific, A-11001)	IF – 1:1000<br>FC – 1:1000
Goat anti-rabbit IgG, Alexa Fluor 546 conjugate (Thermo Fisher Scientific, A-11035)	IF – 1:1000
Goat anti-mouse IgG, Alexa Fluor 546 conjugate (Thermo Fisher Scientific, A-11030)	IF – 1:1000
Anti-rabbit HRP (Cell Signaling Technology, #7074)	WB – 1:1000
Anti-mouse HRP (Sigma-Aldrich, A3682)	WB – 1:1000

Supplementary Table 6: List of primers used in real-time PCR

Gene	Forward sequence	Reverse sequence	Efficacy
HPRT	CCTGGCGTCGTGATTAGTG	ACAGAGGGCTACAATCTGATGG	1.08
***MYH7* (*β-MHC*)**	GCCAAGAGCCGTGACATT	TGCTTTATTCTGCTTCCTCCCA	0.95
***MYH6* (*α-MHC*)**	CAAGAGCCGTGACATTGGTG	TGGCAAGAGTGAGGTTCCC	0.96
TNNI1	TGGATGAGGAGCATACGAC	ATGGACACCTTGTGCTTGGA	1.02
TNNI3	CCTCAAGCAGGTGAAGAAGG	CAGTAGGCAGGAAGGCTCAG	0.96
***MYL2* (*MLC2V*)**	GGCTGATTACGTTCGGGAAATG	CTTCTCCGTGGGTGATGATGTG	0.95
***MYL7* (*MLC2A*)**	CCCCATCAACTTCACCGTCTT	ACTCATCCTTGTTCACCACCC	0.95
OPA1	ACTGGAAGAATCGGACCCAAG	GGTGCTCCTCATTACATTTCAACA	0.95
MFN2	ATGCATCCCCACTTAAGCAC	CCAGAGGGCAGAACTTTGTC	1.03
PPARa	ATGGTGGACACGGAAAGCC	CGATGGATTGCGAAATCTCTTGG	1.14
PPARGC1a	ACGAAGCAGACAAGACCGG	GATTGCGTGCCATCCCAAG	1.21
