# Supplementary material for: Comprehensive promotion of iPSC-CM maturation by integrating metabolic medium with nanopatterning and electrostimulation
Source: Nat Commun. 2025 Mar 21;16:2785. doi: 10.1038/s41467-025-58044-6 (PMC11928738; doi:10.1038/s41467-025-58044-6)
Supplement: Supplementary file 2 — Description of Additional Supplementary Files [file 41467_2025_58044_MOESM2_ESM.pdf]

Description of Additional Supplementary Files

File Name: Supplementary Data 1
Description: GSEA core enrichment genes of downregulated pathway clusters (Figure 7e) in the MM+NP+ES group compared to the MM group.

File Name: Supplementary Data 2
Description: GSEA core enrichment genes of upregulated pathway clusters (Figure 9a) in the MM+NP+ES group compared to the MM group.

File Name: Supplementary Data 3
Description: List of genes used to compare the RNA-seq data from the MM+NP+ES group with the available datasets from heart fetal ventricle and adult heart samples (Supplementary Figure 6).

File Name: Supplementary Movie 1
Description: Movie sequences of iPSC-CMs from the four experimental groups on day 42.
