# Supplementary material for: Comprehensive promotion of iPSC-CM maturation by integrating metabolic medium with nanopatterning and electrostimulation
Source: Nat Commun. 2025 Mar 21;16:2785. doi: 10.1038/s41467-025-58044-6 (PMC11928738; doi:10.1038/s41467-025-58044-6)
Supplement: Supplementary file 7 — Reporting Summary [file 41467_2025_58044_MOESM7_ESM.pdf]

nature portfolio

Corresponding author(s): Kaomei Guan and Mario Schubert

Last updated by author(s): Jan 7, 2025

Reporting Summary

Nature Portfolio wishes to improve the reproducibility of the work that we publish. This form provides structure for consistency and transparency in reporting. For further information on Nature Portfolio policies, see our Editorial Policies and the Editorial Policy Checklist.

Statistics

For all statistical analyses, confirm that the following items are present in the figure legend, table legend, main text, or Methods section.

n/a	Confirmed	
☐	☒	The exact sample size (*n*) for each experimental group/condition, given as a discrete number and unit of measurement
☐	☒	A statement on whether measurements were taken from distinct samples or whether the same sample was measured repeatedly
☐	☒	The statistical test(s) used AND whether they are one- or two-sided<br>*Only common tests should be described solely by name; describe more complex techniques in the Methods section.*
☐	☒	A description of all covariates tested
☐	☒	A description of any assumptions or corrections, such as tests of normality and adjustment for multiple comparisons
☐	☒	A full description of the statistical parameters including central tendency (e.g. means) or other basic estimates (e.g. regression coefficient) AND variation (e.g. standard deviation) or associated estimates of uncertainty (e.g. confidence intervals)
☐	☒	For null hypothesis testing, the test statistic (e.g. *F*, *t*, *r*) with confidence intervals, effect sizes, degrees of freedom and *P* value noted<br>*Give P values as exact values whenever suitable.*
☒	☐	For Bayesian analysis, information on the choice of priors and Markov chain Monte Carlo settings
☒	☐	For hierarchical and complex designs, identification of the appropriate level for tests and full reporting of outcomes
☐	☒	Estimates of effect sizes (e.g. Cohen's *d*, Pearson's *r*), indicating how they were calculated

Our web collection on statistics for biologists contains articles on many of the points above.

Software and code

Policy information about availability of computer code

Data collection	Patchmaster (Heka Elektronik); PatchControlHT (Nanion technologies GmbH); AxIS Navigator (Axion Biosystems); IonWizard core (IonOptix); FusionCapt Advance software (Vilber); ZEN (Carl Zeiss); Seahorse Wave Desktop Software (Agilent); CFX Manager (Bio-Rad); FACS Diva software version 8.0.2 (BD Biosciences); FastQC (https://www.bioinformatics.babraham.ac.uk/projects/fastqc/); featureCounts (v2.0.1); DESeq2 R package (v1.38.3); Kallisto (v0.46.1).
Data analysis	Patchmaster (Heka); FitMaster (Heka); Cardiac Analysis Tool (Axion Biosystems); AxIS Metric Plotting Tool (Axion Biosystems); LabChart 8 software (ADInstruments); IonWizard (IonOptix); FlowJo v10.10 (BD Biosciences); Cell Profiler v4.2.6 (Broad Institute); R Studio v2024.04.2 (Posit Software, PBC); GraphPad Prism v10; GSEA software v.4.3.2 (Broad institute); Cytoscape v3.10.1 (https://cytoscape.org/); FIJI (v.1.54f, ImageJ); FusionCapt Advance software (Vilber); Maia motion analysis software (QuoData–Quality & Statistics GmbH); Excel version 2310 (Microsoft)

For manuscripts utilizing custom algorithms or software that are central to the research but not yet described in published literature, software must be made available to editors and reviewers. We strongly encourage code deposition in a community repository (e.g. GitHub). See the Nature Portfolio guidelines for submitting code & software for further information.

Data

Policy information about availability of data

All manuscripts must include a data availability statement. This statement should provide the following information, where applicable:

- Accession codes, unique identifiers, or web links for publicly available datasets
- A description of any restrictions on data availability
- For clinical datasets or third party data, please ensure that the statement adheres to our policy

RNA-seq data obtained in this study are publicly available at the Gene Expression Omnibus (GEO) under accession number GSE290322 (https://www.ncbi.nlm.nih.gov/geo/query/acc.cgi?acc=GSE290322). Publicly available RNA-seq datasets of human fetal ventricle (GSM1536186, GSM1536187) and human adult heart (GSM1536192, GSM1536193) were obtained from GEO under accession code GSE62913 [https://www.ncbi.nlm.nih.gov/geo/query/acc.cgi?acc=GSE62913].

Research involving human participants, their data, or biological material

Policy information about studies with human participants or human data. See also policy information about sex, gender (identity/presentation), and sexual orientation and race, ethnicity and racism.

Reporting on sex and gender	*Use the terms sex (biological attribute) and gender (shaped by social and cultural circumstances) carefully in order to avoid confusing both terms. Indicate if findings apply to only one sex or gender; describe whether sex and gender were considered in study design; whether sex and/or gender was determined based on self-reporting or assigned and methods used. Provide in the source data disaggregated sex and gender data, where this information has been collected, and if consent has been obtained for sharing of individual-level data; provide overall numbers in this Reporting Summary. Please state if this information has not been collected. Report sex- and gender-based analyses where performed, justify reasons for lack of sex- and gender-based analysis.*
Reporting on race, ethnicity, or other socially relevant groupings	*Please specify the socially constructed or socially relevant categorization variable(s) used in your manuscript and explain why they were used. Please note that such variables should not be used as proxies for other socially constructed/relevant variables (for example, race or ethnicity should not be used as a proxy for socioeconomic status). Provide clear definitions of the relevant terms used, how they were provided (by the participants/respondents, the researchers, or third parties), and the method(s) used to classify people into the different categories (e.g. self-report, census or administrative data, social media data, etc.) Please provide details about how you controlled for confounding variables in your analyses.*
Population characteristics	*Describe the covariate-relevant population characteristics of the human research participants (e.g. age, genotypic information, past and current diagnosis and treatment categories). If you filled out the behavioural & social sciences study design questions and have nothing to add here, write "See above."*
Recruitment	*Describe how participants were recruited. Outline any potential self-selection bias or other biases that may be present and how these are likely to impact results.*
Ethics oversight	*Identify the organization(s) that approved the study protocol.*

Note that full information on the approval of the study protocol must also be provided in the manuscript.

Field-specific reporting

Please select the one below that is the best fit for your research. If you are not sure, read the appropriate sections before making your selection.

☒ Life sciences ☐ Behavioural & social sciences ☐ Ecological, evolutionary & environmental sciences

For a reference copy of the document with all sections, see nature.com/documents/nr-reporting-summary-flat.pdf

Life sciences study design

All studies must disclose on these points even when the disclosure is negative.

Sample size	Sample size for each experiment is indicated in the figure legend. For cell culture and biochemical experiments, sample size was chosen based on previous experiments and comparable studies in literature.
Data exclusions	Only iPSC-CM cultures with high purity were used for the experiments to ensure robustness and reliability of our findings.
Replication	Number of independent experiments and replicates are displayed in the figure captions.
Randomization	Cultures were randomly to experimental groups. Wells for treatment with different drugs in MEA experiments were randomly choosen.
Blinding	For the flow cytometry experiments, samples were randomly assigned with numbers without regard to their experimental groups during the

Blinding	staining process and subsequent measurements, and were categorized into their respective conditions after analysis. Similarly, samples for RNA sequencing were submitted to the Deep Sequencing Facility with identifiers that did not disclose the experimental conditions.

Behavioural & social sciences study design

All studies must disclose on these points even when the disclosure is negative.

Study description	*Briefly describe the study type including whether data are quantitative, qualitative, or mixed-methods (e.g. qualitative cross-sectional, quantitative experimental, mixed-methods case study).*
Research sample	*State the research sample (e.g. Harvard university undergraduates, villagers in rural India) and provide relevant demographic information (e.g. age, sex) and indicate whether the sample is representative. Provide a rationale for the study sample chosen. For studies involving existing datasets, please describe the dataset and source.*
Sampling strategy	*Describe the sampling procedure (e.g. random, snowball, stratified, convenience). Describe the statistical methods that were used to predetermine sample size OR if no sample-size calculation was performed, describe how sample sizes were chosen and provide a rationale for why these sample sizes are sufficient. For qualitative data, please indicate whether data saturation was considered, and what criteria were used to decide that no further sampling was needed.*
Data collection	*Provide details about the data collection procedure, including the instruments or devices used to record the data (e.g. pen and paper, computer, eye tracker, video or audio equipment) whether anyone was present besides the participant(s) and the researcher, and whether the researcher was blind to experimental condition and/or the study hypothesis during data collection.*
Timing	*Indicate the start and stop dates of data collection. If there is a gap between collection periods, state the dates for each sample cohort.*
Data exclusions	*If no data were excluded from the analyses, state so OR if data were excluded, provide the exact number of exclusions and the rationale behind them, indicating whether exclusion criteria were pre-established.*
Non-participation	*State how many participants dropped out/declined participation and the reason(s) given OR provide response rate OR state that no participants dropped out/declined participation.*
Randomization	*If participants were not allocated into experimental groups, state so OR describe how participants were allocated to groups, and if allocation was not random, describe how covariates were controlled.*

Ecological, evolutionary & environmental sciences study design

All studies must disclose on these points even when the disclosure is negative.

Study description	*Briefly describe the study. For quantitative data include treatment factors and interactions, design structure (e.g. factorial, nested, hierarchical), nature and number of experimental units and replicates.*
Research sample	*Describe the research sample (e.g. a group of tagged Passer domesticus, all Stenocereus thurberi within Organ Pipe Cactus National Monument), and provide a rationale for the sample choice. When relevant, describe the organism taxa, source, sex, age range and any manipulations. State what population the sample is meant to represent when applicable. For studies involving existing datasets, describe the data and its source.*
Sampling strategy	*Note the sampling procedure. Describe the statistical methods that were used to predetermine sample size OR if no sample-size calculation was performed, describe how sample sizes were chosen and provide a rationale for why these sample sizes are sufficient.*
Data collection	*Describe the data collection procedure, including who recorded the data and how.*
Timing and spatial scale	*Indicate the start and stop dates of data collection, noting the frequency and periodicity of sampling and providing a rationale for these choices. If there is a gap between collection periods, state the dates for each sample cohort. Specify the spatial scale from which the data are taken*
Data exclusions	*If no data were excluded from the analyses, state so OR if data were excluded, describe the exclusions and the rationale behind them, indicating whether exclusion criteria were pre-established.*
Reproducibility	*Describe the measures taken to verify the reproducibility of experimental findings. For each experiment, note whether any attempts to repeat the experiment failed OR state that all attempts to repeat the experiment were successful.*
Randomization	*Describe how samples/organisms/participants were allocated into groups. If allocation was not random, describe how covariates were controlled. If this is not relevant to your study, explain why.*
Blinding	*Describe the extent of blinding used during data acquisition and analysis. If blinding was not possible, describe why OR explain why blinding was not relevant to your study.*

Did the study involve field work? ☐ Yes ☐ No

Field work, collection and transport

Field conditions	*Describe the study conditions for field work, providing relevant parameters (e.g. temperature, rainfall).*
Location	*State the location of the sampling or experiment, providing relevant parameters (e.g. latitude and longitude, elevation, water depth).*
Access & import/export	*Describe the efforts you have made to access habitats and to collect and import/export your samples in a responsible manner and in compliance with local, national and international laws, noting any permits that were obtained (give the name of the issuing authority, the date of issue, and any identifying information).*
Disturbance	*Describe any disturbance caused by the study and how it was minimized.*

Reporting for specific materials, systems and methods

We require information from authors about some types of materials, experimental systems and methods used in many studies. Here, indicate whether each material, system or method listed is relevant to your study. If you are not sure if a list item applies to your research, read the appropriate section before selecting a response.

Materials & experimental systems

n/a	Involved in the study
☐	☒ Antibodies
☐	☒ Eukaryotic cell lines
☒	☐ Palaeontology and archaeology
☒	☐ Animals and other organisms
☒	☐ Clinical data
☒	☐ Dual use research of concern
☒	☐ Plants

Methods

n/a	Involved in the study
☒	☐ ChIP-seq
☐	☒ Flow cytometry
☒	☐ MRI-based neuroimaging

Antibodies

Antibodies used

Antibody/ Supplier / catalog # / clone/ lot #:

α-Actinin (Sarcomeric) antibody produced in mouse / Sigma Aldrich / A7811
Phalloidin Alexa Fluor 633/ Thermo Scientific / A-22284
RYR2 antibody produced in rabbit / Sigma Aldrich / HAP020028
Mouse Anti-Connexin-43 / BD Bioscience / 610061
Cardiac Troponin T Antibody, anti-human/mouse/rat, REAfinity™/ Miltenyi Biotec / 130-120-543
Troponin T, Cardiac Isoform Ab-1 / Thermo Scientific / MS-295-P1
Ki-67 Antibody, anti-human/mouse, FITC, REAfinity™ / Miltenyi Biotec / 130-117-691
REA Control Antibody, human IgG1, FITC, REAfinity™ / Miltenyi Biotec / 130-113-449
REA Control Antibody, human IgG1, APC, REAfinity™ / Miltenyi Biotec / 130-113-446
GAPDH Antibody (G-9) / Santa-Cruz / sc-365062
SRF (D71A9) XP® Rabbit IgG / Cell Signaling Technology / #5147
Tom20 Antibody (F-10) / Santa Cruz / sc-17764
Goat anti-Mouse IgG (H+L) Cross-Adsorbed Secondary Antibody, Alexa Fluor™ 488 / Thermo Fisher Scientific / A-11001
Goat anti-Rabbit IgG (H+L) Highly Cross-Adsorbed Secondary Antibody, Alexa Fluor™ 546 / Thermo Fisher Scientific / A-11035
Goat anti-Mouse IgG (H+L) Highly Cross-Adsorbed Secondary Antibody, Alexa Fluor™ 546 / Thermo Fisher Scientific / A-11030
Anti-rabbit IgG, HRP-linked Antibody / Cell Signaling Technology / #7074
Anti-mouse HRP / Sigma-Aldrich / A3682

Validation

Validation statements available from manufacturers (antibody /supplier name/ catalog# / validation statement):

Monoclonal Anti-α-Actinin (Sarcomeric) antibody produced in mouse / Sigmaaldrich / A7811 / https://www.sigmaaldrich.com/DE/en/product/sigma/a7811

Phalloidin Alexa Fluor 633/ Thermo Scientific / A-22284
https://www.thermofisher.com/order/catalog/product/de/en/A22284

RYR2 antibody produced in rabbit / Sigma Aldrich / HAP020028 / https://www.sigmaaldrich.com/DE/de/product/sigma/hpa020028

Mouse Anti-Connexin-43 / BD Bioscience / 610061 / https://www.bdbiosciences.com/en-nl/products/reagents/microscopy-imaging-reagents/immunofluorescence-reagents/purified-mouse-anti-connexin-43.610061

Cardiac Troponin T Antibody, anti-human/mouse/rat, REAfinity™/ Miltenyi Biotec / 130-120-543 / https://www.miltenyibiotec.com/

DE-en/products/cardiac-troponin-t-antibody-anti-human-mouse-rat-reafinity-rea400.html#Conjugate=APC:size=30-tests-in-60-uL

Troponin T, Cardiac Isoform Ab-1 / Thermo Scientific / MS-295-P1 / https://www.biocompare.com/Product-Reviews/169115-Cardiac-troponin-T-immunofluorescence-of-human-cardiomyocytes/

Ki-67 Antibody, anti-human/mouse, FITC, REAfinity™ / Miltenyi Biotec / 130-117-691 / https://www.miltenyibiotec.com/DE-en/products/ki-67-antibody-anti-human-mouse-reafinity-rea183.html#Conjugate=FITC:size=100-tests-in-200-uL

REA Control Antibody, human IgG1, FITC, REAfinity™ / Miltenyi Biotec / 130-113-449 / https://www.miltenyibiotec.com/DE-en/products/rea-control-antibody-human-igg1-reafinity-rea293.html#Conjugate=FITC:size=30-ug-in-200-uL

REA Control Antibody, human IgG1, APC, REAfinity™ / Miltenyi Biotec / 130-113-446 / https://www.miltenyibiotec.com/DE-en/products/rea-control-antibody-human-igg1-reafinity-rea293.html#Conjugate=APC:size=30-ug-in-200-uL

GAPDH Antibody (G-9) / Santa-Cruz / sc-365062 / https://www.scbt.com/p/gapdh-antibody-g-9?srsltid=AfmBOorh1zEzautT3GnH42kOxHJThIDdl5Lo0IEnzeqkdw26w1SXpTNQ

SRF (D71A9) XP® Rabbit IgG / Cell Signaling Technology / #5147 / https://www.cellsignal.com/products/primary-antibodies/srf-d71a9-xp-rabbit-mab/5147?srsltid=AfmBOopW2rpsV7Do8TI2ztZs5WpRPWiHPAQexzDCuKcMou5hcNJ5GxQ1

Tom20 Antibody (F-10) / Santa Cruz / sc-17764 / https://www.scbt.com/p/tom20-antibody-f-10?srsltid=AfmBOorDuUgmV2qM6Ih2XE2ClWVlJ4X9Vnw6QWmL0FanC6ggaqwbux9S

Goat anti-Mouse IgG (H+L) Cross-Adsorbed Secondary Antibody, Alexa Fluor™ 488 / Thermo Fisher Scientific / A-11001 / https://www.thermofisher.com/antibody/product/Goat-anti-Mouse-IgG-H-L-Cross-Adsorbed-Secondary-Antibody-Polyclonal/A-11001

Goat anti-Rabbit IgG (H+L) Highly Cross-Adsorbed Secondary Antibody, Alexa Fluor™ 546 / Thermo Fisher Scientific / A-11035 / https://www.thermofisher.com/antibody/product/Goat-anti-Rabbit-IgG-H-L-Highly-Cross-Adsorbed-Secondary-Antibody-Polyclonal/A-11035

Goat anti-Mouse IgG (H+L) Highly Cross-Adsorbed Secondary Antibody, Alexa Fluor™ 546 / Thermo Fisher Scientific / A-11030 / https://www.thermofisher.com/antibody/product/Goat-anti-Mouse-IgG-H-L-Highly-Cross-Adsorbed-Secondary-Antibody-Polyclonal/A-11030

Anti-rabbit IgG, HRP-linked Antibody / Cell Signaling Technology / #7074 / https://www.cellsignal.com/products/secondary-antibodies/anti-rabbit-igg-hrp-linked-antibody / #7074

Anti-mouse HRP / Sigma-Aldrich / A3682 / https://www.sigmaaldrich.com/DE/en/product/sigma/a3682?srsltid=AfmBOooeiwXQs95BOFyUsT7h_GzKxqPJFuXQuq11ntzcclL6E_n6M67O

Eukaryotic cell lines

Policy information about cell lines and Sex and Gender in Research

Cell line source(s)	iWTD2.1 (UMGi001-A.1, FB2-iPS1, female donor) was previously generated from dermal fibroblasts using STEMCCA lentivirus as reported in Streckfuss-Bömeke et al., 2013 (https://doi.org/10.1093/eurheartj/ehs203).<br>iBM76.3 (UMGi005-A.3, MSC3-iPS3, female donor) was previously generated from mesenchymal stem cells using STEMCCA lentivirusas reported in Streckfuss-Bömeke et al., 2013 (https://doi.org/10.1093/eurheartj/ehs203).<br>isWT7.22 (UMGi020-B clone 22, female donor) was previously generated from dermal fibroblasts using the integration-free CytoTune-iPS 2.0 Sendai Reprogramming Kit as reported in Rössler et al., 2021(https://doi.org/10.1002/jbmr.4322).
Authentication	All three cell lines were authenticated by karyotyping and pluripotency assessment, as published in Cyganek et al. 2018 (DOI: 10.1172/jci.insight.99941) and Rössler et al. 2021 (https://doi.org/10.1002/jbmr.4322). Regular mycoplasma testing was conducted via PCR analysis using specific primers (for: 5’-ACACCATGGGAGCTGGTAAT-3’ and rev: 5’-CTTCWTCGACTTYCAGACCCAAGGCAT-3’), confirming the absence of contamination.
Mycoplasma contamination	All cell lines were regularly tested negative for myoplasma contamination (every 2 weeks).
Commonly misidentified lines<br>(See ICLAC register)	*Name any commonly misidentified cell lines used in the study and provide a rationale for their use.*

Palaeontology and Archaeology

Specimen provenance	*Provide provenance information for specimens and describe permits that were obtained for the work (including the name of the issuing authority, the date of issue, and any identifying information). Permits should encompass collection and, where applicable, export.*
Specimen deposition	*Indicate where the specimens have been deposited to permit free access by other researchers.*

Dating methods | *If new dates are provided, describe how they were obtained (e.g. collection, storage, sample pretreatment and measurement), where they were obtained (i.e. lab name), the calibration program and the protocol for quality assurance OR state that no new dates are provided.*

☐ Tick this box to confirm that the raw and calibrated dates are available in the paper or in Supplementary Information.

Ethics oversight | *Identify the organization(s) that approved or provided guidance on the study protocol, OR state that no ethical approval or guidance was required and explain why not.*

Note that full information on the approval of the study protocol must also be provided in the manuscript.

Animals and other research organisms

Policy information about studies involving animals; ARRIVE guidelines recommended for reporting animal research, and Sex and Gender in Research

Laboratory animals | *For laboratory animals, report species, strain and age OR state that the study did not involve laboratory animals.*

Wild animals | *Provide details on animals observed in or captured in the field; report species and age where possible. Describe how animals were caught and transported and what happened to captive animals after the study (if killed, explain why and describe method; if released, say where and when) OR state that the study did not involve wild animals.*

Reporting on sex | *Indicate if findings apply to only one sex; describe whether sex was considered in study design, methods used for assigning sex. Provide data disaggregated for sex where this information has been collected in the source data as appropriate; provide overall numbers in this Reporting Summary. Please state if this information has not been collected. Report sex-based analyses where performed, justify reasons for lack of sex-based analysis.*

Field-collected samples | *For laboratory work with field-collected samples, describe all relevant parameters such as housing, maintenance, temperature, photoperiod and end-of-experiment protocol OR state that the study did not involve samples collected from the field.*

Ethics oversight | *Identify the organization(s) that approved or provided guidance on the study protocol, OR state that no ethical approval or guidance was required and explain why not.*

Note that full information on the approval of the study protocol must also be provided in the manuscript.

Clinical data

Policy information about clinical studies

All manuscripts should comply with the ICMJE guidelines for publication of clinical research and a completed CONSORT checklist must be included with all submissions.

Clinical trial registration | *Provide the trial registration number from ClinicalTrials.gov or an equivalent agency.*

Study protocol | *Note where the full trial protocol can be accessed OR if not available, explain why.*

Data collection | *Describe the settings and locales of data collection, noting the time periods of recruitment and data collection.*

Outcomes | *Describe how you pre-defined primary and secondary outcome measures and how you assessed these measures.*

Dual use research of concern

Policy information about dual use research of concern

Hazards

Could the accidental, deliberate or reckless misuse of agents or technologies generated in the work, or the application of information presented in the manuscript, pose a threat to:

No	Yes	
☒	☐	Public health
☒	☐	National security
☒	☐	Crops and/or livestock
☒	☐	Ecosystems
☒	☐	Any other significant area

Experiments of concern

Does the work involve any of these experiments of concern:

No	Yes	
☒	☐	Demonstrate how to render a vaccine ineffective
☒	☐	Confer resistance to therapeutically useful antibiotics or antiviral agents
☒	☐	Enhance the virulence of a pathogen or render a nonpathogen virulent
☒	☐	Increase transmissibility of a pathogen
☒	☐	Alter the host range of a pathogen
☒	☐	Enable evasion of diagnostic/detection modalities
☒	☐	Enable the weaponization of a biological agent or toxin
☒	☐	Any other potentially harmful combination of experiments and agents

Plants

Seed stocks: -

Novel plant genotypes: -

Authentication: -

ChIP-seq

Data deposition

☐ Confirm that both raw and final processed data have been deposited in a public database such as GEO.

☐ Confirm that you have deposited or provided access to graph files (e.g. BED files) for the called peaks.

Data access links
May remain private before publication.
For "Initial submission" or "Revised version" documents, provide reviewer access links. For your "Final submission" document, provide a link to the deposited data.

Files in database submission
Provide a list of all files available in the database submission.

Genome browser session
(e.g. UCSC)
Provide a link to an anonymized genome browser session for "Initial submission" and "Revised version" documents only, to enable peer review. Write "no longer applicable" for "Final submission" documents.

Methodology

Replicates
Describe the experimental replicates, specifying number, type and replicate agreement.

Sequencing depth
Describe the sequencing depth for each experiment, providing the total number of reads, uniquely mapped reads, length of reads and whether they were paired- or single-end.

Antibodies
Describe the antibodies used for the ChIP-seq experiments; as applicable, provide supplier name, catalog number, clone name, and lot number.

Peak calling parameters
Specify the command line program and parameters used for read mapping and peak calling, including the ChIP, control and index files used.

Data quality
Describe the methods used to ensure data quality in full detail, including how many peaks are at FDR 5% and above 5-fold enrichment.

Software
Describe the software used to collect and analyze the ChIP-seq data. For custom code that has been deposited into a community repository, provide accession details.

Flow Cytometry

Plots

Confirm that:

☒ The axis labels state the marker and fluorochrome used (e.g. CD4-FITC).

☒ The axis scales are clearly visible. Include numbers along axes only for bottom left plot of group (a 'group' is an analysis of identical markers).

☒ All plots are contour plots with outliers or pseudocolor plots.

☒ A numerical value for number of cells or percentage (with statistics) is provided.

Methodology

Sample preparation	Samples were prepared as described in methods section:<br><br>Cells were singularised using collagenase and trypsin, fixed in 4% paraformaldehyde (PFA) for 20 min at RT and stored in PBS containing 1% BSA at 4°C. For staining, iPSC-CMs were permeabilised in PBS containing 1% BSA and 0.1% Triton-X for 10 min at RT. Staining was performed with specific antibodies (Supplementary Table 5). cTNT was detected using either mouse anti-cTNT (Thermo Fisher Scientific, MS-295-P1) or directly coupled cTNT-APC (Miltenyi Biotec, 130-120-543). For cTnT and Tom20 staining, cTNT-APC and anti-Tom20 (Santa Cruz, sc-17764) antibodies were used. Negative controls were performed using either the respective secondary antibodies (for samples detected with the non-coupled primary antibodies) or isotype controls (for samples detected with the directly coupled primary antibodies). After incubation with primary antibodies, cells were washed with PBS containing 1% BSA, followed by incubation with secondary antibodies, and Hoechst 33342 (5 µg/mL). To assess EdU-incorporation, PFA-fixed iPSC-CMs were incubated with mouse anti-cTNT antibody in Click-iT™ permeabilisation and wash reagent (Thermo Fischer Scientific, C10645) overnight, EdU click reaction was performed according to manufacturer's instructions, and DNA was stained with Draq5 (abcam, ab108410, 10 µM). To determine the activity of Ki67, iPSC-CMs were stained with antibodies cTNT-APC and Ki67-FITC (Miltenyi Biotec, 130-117-691) for 1 h at 4°C. DNA was stained with Hoechst 33342. Afterwards, cells were resuspended in PBS containing 1% BSA and analysed on an LSRII or FACS Canto II flow cytometer using FACSDiva software version 8.0.2 (BD Biosciences). At least 10,000 events were recorded for each sample. Flow cytometry data were then analysed using FlowJo v10.10 (BD Biosciences).
Instrument	LSRII or Canto II
Software	Samples were acquired using the BDFACS Diva software version 8.0.2. Flow cytometry data were analyzed using FlowJo version 10.10 (BD).
Cell population abundance	iPSC-CM cultures used in this study had a high purity of >85% cTNT-positive cells.
Gating strategy	The gating strategy is shown in Supplementary Fig. 3. Cells were gated based on FSC-A vs. SSC-A, followed by gating of single cells based on FSC-A vs. FSC-H. Next, residual debris were excluded by gating of the Hoechst33342-positive cell population. Finally, iPSC-CMs were defined as the cTNT-positive population.<br>For EdU-measurements, cells were gated based on FSC-A vs. SSC-A, followed by gating of single cells based on FSC-A vs. FSC-H, and selection of cTNT-positive population.

☒ Tick this box to confirm that a figure exemplifying the gating strategy is provided in the Supplementary Information.

Magnetic resonance imaging

Experimental design

Design type	*Indicate task or resting state; event-related or block design.*
Design specifications	*Specify the number of blocks, trials or experimental units per session and/or subject, and specify the length of each trial or block (if trials are blocked) and interval between trials.*
Behavioral performance measures	*State number and/or type of variables recorded (e.g. correct button press, response time) and what statistics were used to establish that the subjects were performing the task as expected (e.g. mean, range, and/or standard deviation across subjects).*

Acquisition

Imaging type(s) — *Specify: functional, structural, diffusion, perfusion.*

Field strength — *Specify in Tesla*

Sequence & imaging parameters — *Specify the pulse sequence type (gradient echo, spin echo, etc.), imaging type (EPI, spiral, etc.), field of view, matrix size, slice thickness, orientation and TE/TR/flip angle.*

Area of acquisition — *State whether a whole brain scan was used OR define the area of acquisition, describing how the region was determined.*

Diffusion MRI ☐ Used ☐ Not used

Preprocessing

Preprocessing software — *Provide detail on software version and revision number and on specific parameters (model/functions, brain extraction, segmentation, smoothing kernel size, etc.).*

Normalization — *If data were normalized/standardized, describe the approach(es): specify linear or non-linear and define image types used for transformation OR indicate that data were not normalized and explain rationale for lack of normalization.*

Normalization template — *Describe the template used for normalization/transformation, specifying subject space or group standardized space (e.g. original Talairach, MNI305, ICBM152) OR indicate that the data were not normalized.*

Noise and artifact removal — *Describe your procedure(s) for artifact and structured noise removal, specifying motion parameters, tissue signals and physiological signals (heart rate, respiration).*

Volume censoring — *Define your software and/or method and criteria for volume censoring, and state the extent of such censoring.*

Statistical modeling & inference

Model type and settings — *Specify type (mass univariate, multivariate, RSA, predictive, etc.) and describe essential details of the model at the first and second levels (e.g. fixed, random or mixed effects; drift or auto-correlation).*

Effect(s) tested — *Define precise effect in terms of the task or stimulus conditions instead of psychological concepts and indicate whether ANOVA or factorial designs were used.*

Specify type of analysis: ☐ Whole brain ☐ ROI-based ☐ Both

Statistic type for inference (See Eklund et al. 2016) — *Specify voxel-wise or cluster-wise and report all relevant parameters for cluster-wise methods.*

Correction — *Describe the type of correction and how it is obtained for multiple comparisons (e.g. FWE, FDR, permutation or Monte Carlo).*

Models & analysis

n/a	Involved in the study
☐	☐ Functional and/or effective connectivity
☐	☐ Graph analysis
☐	☐ Multivariate modeling or predictive analysis

Functional and/or effective connectivity — *Report the measures of dependence used and the model details (e.g. Pearson correlation, partial correlation, mutual information).*

Graph analysis — *Report the dependent variable and connectivity measure, specifying weighted graph or binarized graph, subject- or group-level, and the global and/or node summaries used (e.g. clustering coefficient, efficiency, etc.).*

Multivariate modeling and predictive analysis — *Specify independent variables, features extraction and dimension reduction, model, training and evaluation metrics.*
